# Visual crowding is unaffected by adaptation-induced spatial compression

**DOI:** 10.1167/18.3.12

**Published:** 2018-03-23

**Authors:** Alison Chambers, Alan Johnston, Neil W. Roach

**Affiliations:** chambers.alc@gmail.com; alan.johnston@nottingham.ac.uk https://www.nottingham.ac.uk/psychology/people/alan.johnston; neil.roach@nottingham.ac.uk https://www.nottingham.ac.uk/psychology/people/neil.roach; Visual Neuroscience Group, School of Psychology, The University of Nottingham, Nottingham, UK; Visual Neuroscience Group, School of Psychology, The University of Nottingham, Nottingham, UK; Visual Neuroscience Group, School of Psychology, The University of Nottingham, Nottingham, UK

**Keywords:** *crowding*, *adaptation*, *aftereffects*

## Abstract

It has recently been shown that adapting to a densely textured stimulus alters the perception of visual space, such that the distance between two points subsequently presented in the adapted region appears reduced (Hisakata, Nishida, & Johnston, 2016). We asked whether this form of adaptation-induced spatial compression alters visual crowding. To address this question, we first adapted observers to a dynamic dot texture presented within an annular region surrounding the test location. Following adaptation, observers perceived a test array comprised of multiple oriented dot dipoles as spatially compressed, resulting in an overall reduction in perceived size. We then tested to what extent this spatial compression influences crowding by measuring orientation discrimination of a single dipole flanked by randomly oriented dipoles across a range of separations. Following adaptation, we found that the magnitude of crowding was predicted by the physical rather than perceptual separation between center and flanking dipoles. These findings contrast with previous studies in which crowding has been shown to increase when motion-induced position shifts act to reduce apparent separation (Dakin, Greenwood, Carlson, & Bex, 2011; Maus, Fischer, & Whitney, 2011).

## Introduction

A fundamental task of our visual system is to determine the location of objects within the visual field. The spatial arrangement of objects in a visual scene is reflected in the pattern of light projected onto the retina. Photoreceptors at adjacent locations on the retinal surface respond to neighboring points in the scene, thus forming a systematic sampled representation, within which the relative arrangement of objects in the visual scene is preserved. Because connections between neurons in successive visual areas are topographically organized, this mapping of visual space is maintained throughout the visual pathway (Wandell, Dumoulin, & Brewer, 2007; Wandell & Winawer, 2011). Given the highly specialized retinotopic organization of the visual system, one might think establishing the spatial location and size of an object is a computationally simple process, deduced directly from the location of the responsive neurons on the visual field map. However, under some conditions, systematic biases occur between the perceived and physical spatial arrangement of objects in a visual scene.

Some of the most well-known distortions of spatial position and size involve moving objects (Kirschfeld & Kammer, 1999; Nijhawan, 1994; Whitney & Cavanagh, 2000). For example, the presence of motion within a stationary contrast envelope (e.g., a drifting Gabor patch) induces a shift in perceived location in the direction of the stimulus motion (De Valois & De Valois, 1991). In the flash-drag effect, the position of a stationary flashed stimulus near to a moving stimulus can appear shifted in the direction of the moving stimulus (Durant & Johnston, 2004; Whitney & Cavanagh, 2000). Similarly, in the flash-lag effect a moving stimulus can appear further ahead of an adjacent flashed stimulus (Nijhawan, 1994). Furthermore, motion adaptation aftereffects can change the perceived size of an object; for example, adaptation to a contracting spiral can make a subsequently static stimulus appear to expand (Thompson, 1880).

The spatial context in which objects are presented can also have a profound influence on the perceived size of an object (Westheimer, 2008). For instance, in the classic Ebbinghaus illusion, the perceived size of a circular disk is modulated by the relative size of nearby disks. The physical representation of the center circle remains unchanged; however, the context surrounding the object alters its perceived size. Similarly, the perceived size of an object is not entirely dependent upon its retinal representation, but also on its apparent distance, a phenomenon referred to as size constancy (Emmert, 1881).

The wealth of studies demonstrating mismatches between the perceived location and/or size of objects and their physical retinal representation suggests that the spatial mapping of visual inputs is not solely dependent on the spatial arrangement of light falling onto the retina. Instead, these findings demonstrate that the encoding of size and position by the visual system is flexible and modulated by subsequent stages of visual processing.

Köhler and Wallach (1944) were the first to use adaptation to a static stimulus to manipulate apparent size in their seminal work on figural aftereffects. A classic example of a figural aftereffect is where adaptation to a circle results in a subsequently viewed smaller circle to appear reduced in size. Evidence from neuroimaging has shown changes in the perceived size of an object resulting from this form of figural aftereffect can coincide with changes in the extent of blood oxygen level-dependent signal in area V1. These changes were attributed to low-level effects of contrast gain control, with neurones responding to the test stimulus being modulated by the local inhibition induced by the prior adapting stimulus (Pooresmaeili, Arrighi, Biagi, & Morrone, 2013). More recently, aftereffects have also been shown for judgments of the mean size of a group of elements (Corbett, Wurnitsch, Schwartz, & Whitney, 2012). These distortions cannot be easily accounted for on the basis of low-level contrast-gain processes and may reflect the effect of adaptation on representations of the summary statistics of element size (Corbett & Melcher, 2014; Corbett et al., 2012).

In a recent study, Hisakata et al. (2016) showed adaptation to a dense texture reduced the perceived separation of two subsequently presented dots. This spatial compression was present when the adapting texture encompassed the entire viewing field, ruling out the possibility that it could be caused by adaptation to the larger-sized adapting stimulus. They also showed the opposite was true for the perceived density of a texture, wherein following adaptation, the perceived density of an equally dense texture appeared sparser. These findings indicated the spatial compression resulting from adaptation could not be accounted for by the low-level differences in the spatial frequency of the stimuli or by a size-sensitive mechanism. Interestingly, these results suggest adaptation to dense textures alters the internal mapping of space in the visual system.

In this paper we show that adaptation to an annular texture induces spatial compression in the region previously surrounded by the annulus; individual elements appear compressed together producing an overall reduction in the perceived size of the test stimulus. It is currently unclear whether, when an array of elements appears spatially compressed following adaptation, this distortion affects visual sensitivity to variation in the individual elements comprising it. Therefore, in the second part of this paper, we determine if the ability to discriminate the orientation of a single dipole element within the array is altered following adaptation-induced spatial compression.

It is well known that when flanking stimuli surround a target the ability to distinguish the properties of the target are degraded, an effect often referred to as crowding (Levi, 2008). The distance between the test and flanking stimuli is a critical factor determining the extent by which performance is affected; flankers only impede performance when they are separated from the test by less than 0.4–0.5 times the eccentricity (Bouma, 1970). Therefore, we may anticipate that under conditions in which the perception of visual space is compressed and individual elements appear closer together, the ability to discriminate the orientation of a single element may be reduced.

Two similar studies have previously measured performance on an orientation discrimination task when the position of the flanking stimuli appeared either closer to or further away from the target (Dakin et al., 2011; Maus et al., 2011). In both of these studies, the perceived location of the flanking stimuli were manipulated by altering the motion direction of the flankers (De Valois & De Valois, 1991). They found performance was determined by the perceived location of the flankers and not their physical location. Surprisingly, our results showed the opposite pattern. Following adaptation-induced spatial compression performance was dependent on the physical location of the stimuli and not the perceived position.

## Methods

### Observers

One of the authors (ALC) and three experienced psychophysical observers who were naive to the purpose of the study took part. All observers had normal or corrected-to-normal vision.

### Apparatus

The stimuli were presented on a gamma-corrected NEC MultiSync FP1370 CRT monitor (NEC, Itasca, IL) at a frame rate of 100 Hz. The resolution of the display was 1,280 × 1,026 pixels with 1 pixel subtending 1 arcmin at a viewing distance of 103 cm. Observers viewed the stimuli binocularly in a darkened room and with their head placed in a chin rest for stability. Experiments were run in MATLAB (MathWorks, Natick, MA) using elements of the Psychophysics Toolbox (Brainard, 1997; Kleiner, Brainard, & Pelli, 2007).

### Stimuli

**Figure 1:**
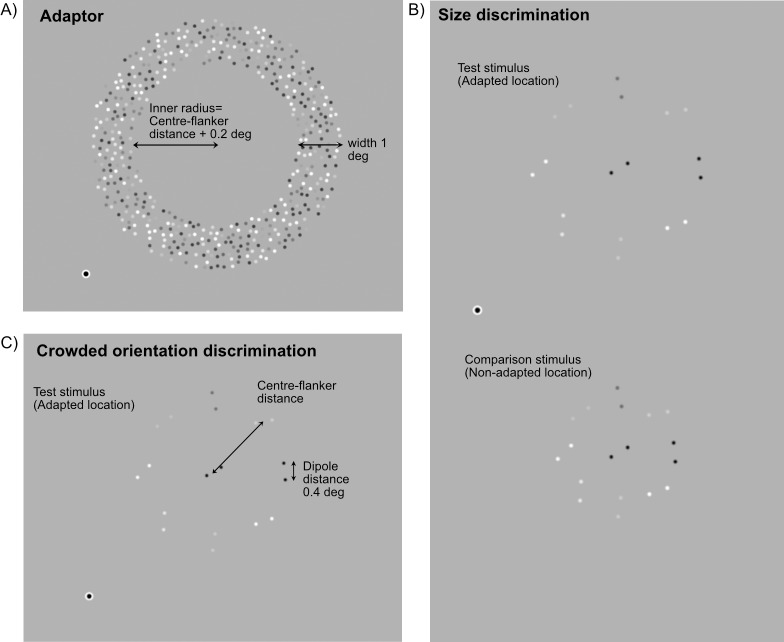
Schematic of the stimuli used in the size discrimination and crowded orientation discrimination tasks. (A) For both tasks, the adapting stimulus consisted of 5 Hz flickering dots presented within an annular region. (B) In the size discrimination task, observers compared the spatial extent of two arrays of oriented dot dipoles—a test stimulus positioned within the annular adapting region and a comparison stimulus presented at an unadapted location. (C) In the crowded orientation discrimination task, observers were asked to indicate if the central dipole of a test stimulus presented at the adapted location was tilted clockwise or counterclockwise relative to vertical. Crowding was induced by randomly oriented flanker dipoles.

Figure 1 shows a schematic of the adapting and test stimuli used in the size and orientation discrimination tasks. Observers were instructed to maintain fixation on a white fixation dot (12 arcmin in diameter) presented in the center of the screen.

The adapting stimulus was the same in both tasks and consisted of a ring of flickering dots with a mean density of 33 dots/deg^2^. The adapting stimulus occupied the region surrounding the test stimulus and did not overlap the test. We chose to use this particular arrangement to minimize any reduction in the perceived contrast of the test stimulus and/or changes in perceived position due to asymmetric contrast adaptation (Whitaker, McGraw, & Levi, 1997). The inner radius of the adapting stimulus was 0.2° greater than the radius of the test stimulus and the outer radius was 1° greater than the inner radius. The luminance of each dot was sinusoidally modulated at a frequency of 5 Hz and at 80% Michelson contrast. The starting phase of each dot comprising the adapter was chosen at random. The adapting stimulus was presented 3° to the right and either 3° above or below fixation (a visual eccentricity of 4.24°).

The test stimulus for both the size and orientation discrimination tasks was an array of oriented dot dipoles, comprising a single dipole positioned at the center of the adapted region and eight flanking dipoles arranged in a circular configuration (positions evenly spaced and fixed relative to cardinal axes). The dots forming each dipole were 5 arcmin in diameter and separated by 0.4°. The orientation of the central dipole was randomized on each trial of the size discrimination task, but systematically manipulated in the orientation discrimination task. Flanking dipole orientations were randomized for both tasks. Test dot luminance was sinusoidally modulated over time at a frequency of 5 Hz with 100% peak Michelson contrast. The temporal modulation of each dot was in-phase with its dipole partner, but starting phases were randomly allocated across dipoles.

In the size discrimination task, a comparison stimulus was also presented on the opposite side of the horizontal meridian to the test (i.e., 3° to the right and either 3° above or below fixation). The comparison consisted of nine dipoles, all of which were randomly oriented.

### Procedure

#### Size discrimination task

Adaptation-induced changes in the perceived size of the test stimulus were measured using a spatial two-alternative forced choice and method of constants procedure. The adapting stimulus appeared for 30 s on the first trial and 5 s on subsequent trials. Following a 500 ms blank interstimulus interval, the test and comparison stimuli appeared for 500 ms, followed by a brief interstimulus interval of 100 ms. Observers were instructed to indicate via a button press the set of dipoles that appeared larger (top or bottom). The next trial commenced 100 ms after the observer had made their response.

In each run, the distance between center and flanking dipoles in the test stimulus was set to one of seven values ranging from 1.27° to 2.55° while the center-flanking distance of the comparison stimulus was manipulated via a method of constant stimuli (nine log-spaced levels, offset relative to the test to ensure adequate coverage of the psychometric function). Each run consisted of 90 trials, with 10 repetitions for each comparison stimulus. Observers completed four runs per test stimulus condition (2,520 total trials).

#### Orientation discrimination task (crowding)

We measured orientation discrimination thresholds for a single dipole presented alone (no crowding) and in the presence of flanking dipoles (crowding), with and without adaptation. In all experimental conditions, the task was to indicate via a button press if the center dipole of the test stimulus appeared oriented clockwise or counterclockwise of vertical. The orientation of the center dipole was varied according to the method of constant stimuli (nine levels, evenly spaced in 2.5° increments around vertical). The step size was increased to 5° for observer ALC. In separate runs, center-flanking distance was systematically varied between 1.27° and 2.55°. Data were also collected for an uncrowded condition in which only the center dipole was presented.

The adaptation procedure was identical to that used in the size discrimination experiment. Following the adaptation period there was a brief interstimulus interval of 500 ms and the test stimulus appeared in the same location as the adaptor for 500 ms and observers made their response. Due to differences in the magnitude of crowding in upper and lower visual field locations (He, Cavanagh, & Intriligator, 1996, 1997), the vertical location of the adapting stimulus was varied across runs, with equal number of runs in the upper and lower field. In the no adaption conditions, the vertical position of the test stimulus was randomly chosen on each trial.

While both the size of each dot and the separation between dots within a dipole were kept constant in the main crowding study, we also ran a control, unadapted experiment in which we manipulated these factors. Participants were first retested using the original approach at center-flanking distances of 1.91° and 1.27°. The change between these two conditions equates to a reduction in center-flanking distance of 33.5%. Two additional versions of the 1.27° condition were then run in which the: (a) intra-dipole separation (0.4° to 0.267°) or (b) intra-dipole separation (0.4° to 0.267°) and dot size (5 to 3.33 pixels) were reduced by the same factor.

Each run consisted of 90 trials, with 10 presentations of each orientation randomly ordered within a run. Observers completed 4–6 runs per experimental condition. Six additional runs were completed for the no-adaptation baseline condition (no crowding). Data were accumulated over several experimental sessions and different conditions were completed in a pseudorandom order.

### Analysis

Psychometric functions were constructed for each condition and fitted with a logistic of the form:

\newcommand{\bialpha}{\boldsymbol{\alpha}}\newcommand{\bibeta}{\boldsymbol{\beta}}\newcommand{\bigamma}{\boldsymbol{\gamma}}\newcommand{\bidelta}{\boldsymbol{\delta}}\newcommand{\bivarepsilon}{\boldsymbol{\varepsilon}}\newcommand{\bizeta}{\boldsymbol{\zeta}}\newcommand{\bieta}{\boldsymbol{\eta}}\newcommand{\bitheta}{\boldsymbol{\theta}}\newcommand{\biiota}{\boldsymbol{\iota}}\newcommand{\bikappa}{\boldsymbol{\kappa}}\newcommand{\bilambda}{\boldsymbol{\lambda}}\newcommand{\bimu}{\boldsymbol{\mu}}\newcommand{\binu}{\boldsymbol{\nu}}\newcommand{\bixi}{\boldsymbol{\xi}}\newcommand{\biomicron}{\boldsymbol{\micron}}\newcommand{\bipi}{\boldsymbol{\pi}}\newcommand{\birho}{\boldsymbol{\rho}}\newcommand{\bisigma}{\boldsymbol{\sigma}}\newcommand{\bitau}{\boldsymbol{\tau}}\newcommand{\biupsilon}{\boldsymbol{\upsilon}}\newcommand{\biphi}{\boldsymbol{\phi}}\newcommand{\bichi}{\boldsymbol{\chi}}\newcommand{\bipsi}{\boldsymbol{\psi}}\newcommand{\biomega}{\boldsymbol{\omega}}\begin{equation}\tag{1}p = {1 \over {1 + {e^{{{PSE - X} \over {JND}}}}}}\end{equation}

where *p* is the proportion of “comparison larger” (size discrimination task) or “clockwise” (orientation discrimination task), *PSE* is the point of subjective equality and *JND* is the just noticeable difference, or discrimination threshold. Parameter fitting was carried out in MATLAB (MathWorks), using *fmincon* to minimize the negative log-likelihood. The PSE in the size discrimination task was subtracted from the flanker distance of the test stimulus. Therefore, PSE values below zero represented a reduction in the perceived size of the stimulus and positive values an increase in size. The confidence intervals associated with each PSE and threshold estimate were obtained via nonparametric bootstrapping (Efron & Tibshirani, 1993).

## Results

### Adaptation reduces perceived size

**Figure 2:**
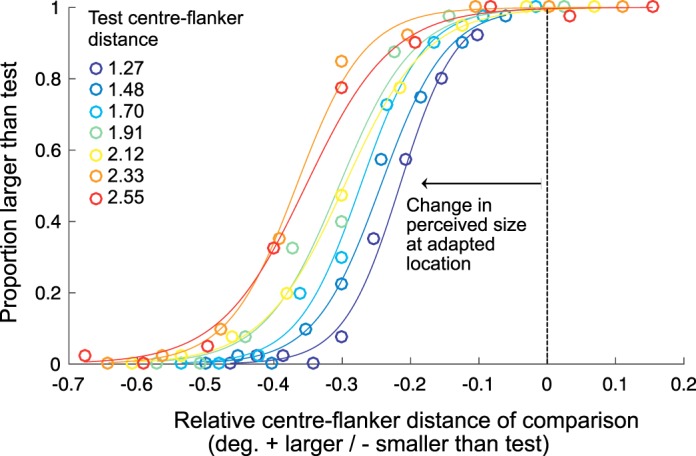
Raw data and psychometric fits for one observer (BBB) from the size comparison experiment. The x-axis shows the difference between the center-flanker distance of the comparison (nonadapted location, center-flanker distance varied) and test (adapted location, fixed center-flanker distance) stimuli. The y-axis shows the proportion of trials on which the comparison stimulus was perceived as larger than the test. A shift in the PSE to left of zero (negative) represents a perceived reduction in the size of the stimulus following adaptation. The separate psychometric functions represent data collected from seven different test center-flanker distances. Individual psychometric functions for all observers can be found in Appendix A.

Figure 2 shows example size discrimination data for a naïve observer (BBB). Data points represent the proportion of times the observer judged the size of the comparison stimulus to be larger than the test. Zero on the *x*-axis represents when the comparison (nonadapted) and test (adapted) stimuli have the same center-flanker distance, negative values on the *x*-axis indicate the comparison stimulus was judged to be smaller than the test (adapted) stimulus. In each condition, the point of subjective equality is shifted to the left demonstrating that the observer perceived the stimulus presented at the adapted location to be smaller than its physical size.

**Figure 3:**
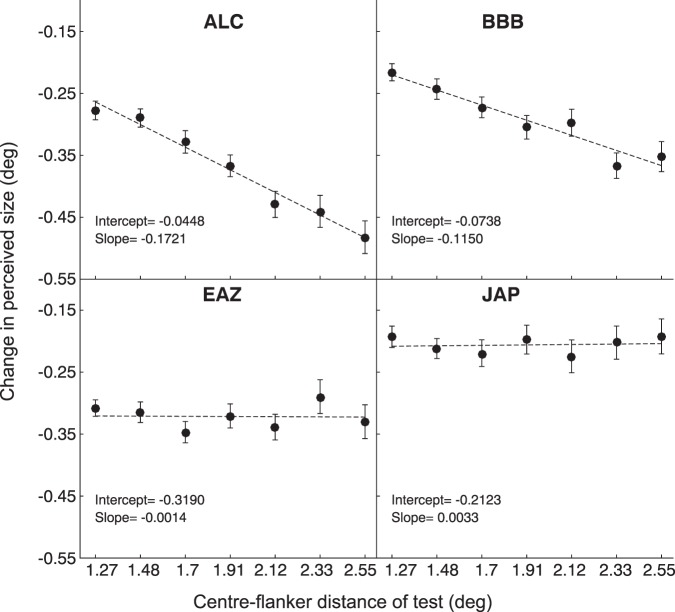
Changes in perceived size following adaptation. Points of subjective equality estimates shown for each observer, plotted as a function of the test stimulus center-flanker distance. In each case, a linear regression is shown, with best-fitting slope and intercept values located in the bottom left of each plot. Error bars indicate 95% bootstrapped confidence intervals.

Figure 3 shows points of subjective equality, plotted as a function of the test center-flanker separation. Robust adaptation-induced reductions in perceived size were found for all observers, as evidenced by the negative shifts in PSE in each condition. Dashed lines show the best fitting linear functions for each observer. For observers ALC and BBB, the magnitude of this compression scaled with stimulus size, approximating a constant percentage of the center-flanker separation. (∼20% for ALC and ∼15% for BBB). However, this is not the case for observers JAP and EAZ, for whom size reductions (measured in degrees) were invariant to changes in center-flanker distance (∼0.3° and ∼0.18° for EAZ and JAP, respectively).

### Crowding is unaffected by spatial compression

**Figure 4:**
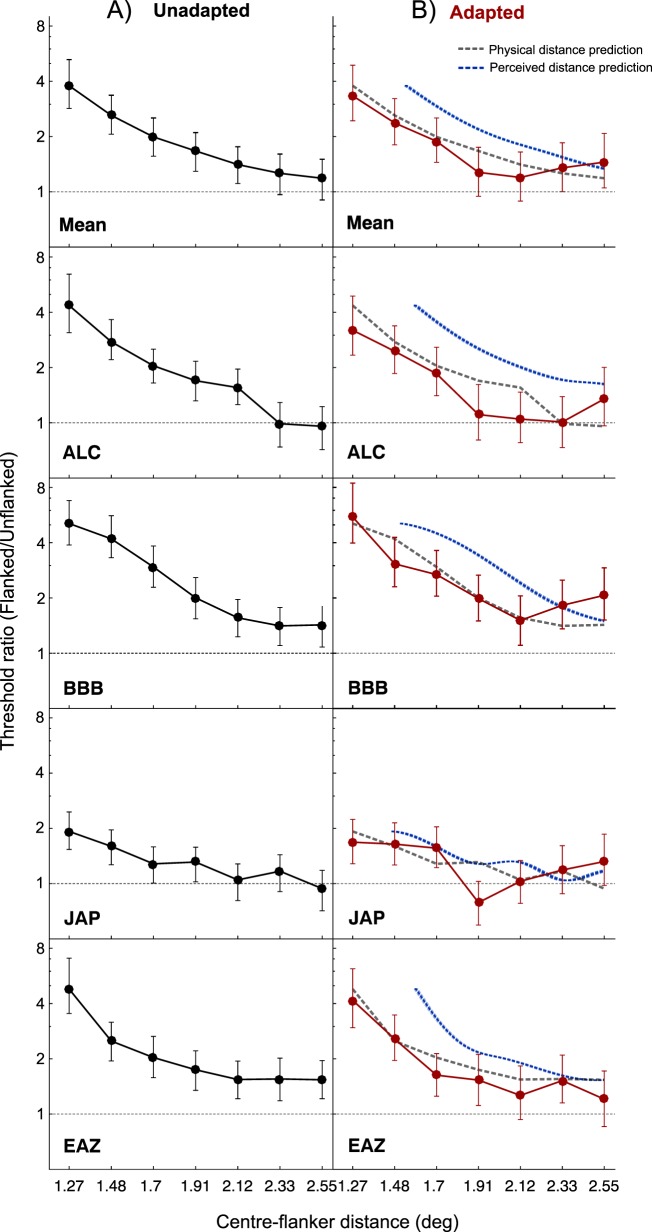
Crowding on the orientation discrimination task, as a function of center-flanker distance. On the left, plotted in black, is performance with no prior adaptation. On the right, in red, is performance following adaptation. For comparison performance without adaptation is plotted in gray, and in blue, the predicted performance based on the perceived location of the flankers following adaptation is plotted. Error bars indicate 95% bootstrapped confidence intervals. Individual psychometric functions for each condition of the orientation discrimination task are shown in Appendix B.

Figure 4A shows the performance of the four observers on the orientation discrimination task in the absence of adaptation. Performance is expressed as the ratio of orientation discrimination thresholds obtained in flanked and unflanked conditions; ratios greater than one indicate crowding. As expected, flanked performance deteriorates with decreasing center-flanker distance. Inspection of the mean threshold ratios and bootstrapped confidence intervals indicates that significant crowding occurred for center-flanker distances of 2.12° or less. This equates to distances less than approximately 50% of the target eccentricity, in agreement with previous crowding studies (e.g., Rosen, Chakravarthi, & Pelli, 2014; Whitney & Levi, 2011).

Performance on the orientation discrimination task following adaptation is plotted in Figure 4B (red symbols). For comparison, gray dashed lines show predictions based on crowding following physical center-flanker separation (replotted from Figure 4A), whereas the blue dashed lines show predictions based on perceived center-flanker separation. The latter set of predictions was obtained for each observer by calculating a cubic spline interpolation of their unadapted crowding function, and linearly transforming the *x*-axis to match variations in perceived center-flanker separation following adaptation. Because adaptation reduces the apparent separation between center and flanker dipoles, one might expect the crowding function to shift to the right. However, this was not the case. No systematic differences in the magnitude of crowding were found between adapted and unadapted conditions, suggesting that crowding is determined by the physical (rather than perceived) center-flanker separation.

### Scaling the dimensions of the stimulus has no influence on orientation discrimination thresholds

In the previous experiment, we quantified adaptation-induced spatial compressions by asking observers to make comparative size judgments between stimuli presented at adapted and unadapted locations of space. Whenever this approach is taken, choices need to be made about how to manipulate the stimuli to best mimic and null the perceptual distortion. In this case, we chose to vary the spatial separation between dipoles, while leaving the size of the dots and the intradipole separation fixed. However, it is possible that adaptation could induce a more uniform rescaling of visual space, affecting one or both of these factors in addition to center-flanker separation. If either of these additional size manipulations acted to reduce crowding, this could offset the effect of spatial compression and explain why adaptation had minimal effect on crowding.

**Figure 5:**
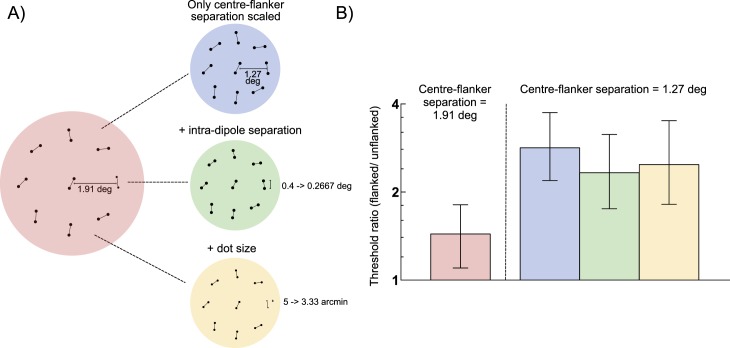
Crowding induced by different forms of dipole array spatial scaling. Mean threshold ratios (flanked/unflanked) for four observers are shown. The red bar indicates crowding with a center-flanker separation of 1.91°, whereas the other three bars indicate crowding with a center-flanker separation of 1.27°. The reduction in center-flanker distance increases crowding, regardless of whether this manipulation occurs in isolation (blue bar), or with concomitant changes in intradipole separation (green bar) or dot size (yellow bar). Error bars indicate 95% confidence intervals.

To test this possibility, we measured orientation discrimination thresholds from the same four observers using different scaled versions of the stimuli. Figure 5A depicts the four different conditions examined. In the original experiment performance on the orientation task was measured as a function of center-flanker separation. We re-collected data for two points on this function: one where little or no crowding occurs (A: 1.91° separation, red disk) and another where the effect of crowding is strong (B: 1.27°, blue disk). To the strong crowding configuration, we then introduced scaling of either the intradipole separation (C: green disk) or intradipole separation and dot size (D: yellow disk) that was proportional to the change in center-flanker separation. Figure 5B plots the magnitude of crowding observed in the different conditions. Scaling the dipole separation and the dot size led to a small, but nonsignificant decrease in the magnitude of crowding (B vs. C, *p* = 0.3069; B vs. D, *p* = 0.5224). This slight decrease in the magnitude of crowding is unlikely to alter the crowding function following adaptation to the extent that the magnitude of crowding corresponds with the perceived location of the flankers.

## Discussion

Our results reveal a dissociation between the effects of adaptation on spatial appearance and discriminability. Adapting to a random dot texture in the region surrounding a set of dot pairs induces a spatial compression effect, wherein the dot pairs appear shifted inward toward each other, reducing perceived size. However, this reduction in apparent separation between central and flanking dot pairs does not affect crowding, as measured with orientation discrimination.

The spatial compression effect we observe shares similarities with previous research on artificial scotomas (Kapadia, Gilbert, & Westheimer, 1994; Tailby & Metha, 2004). In these studies, observers were exposed to large field, dynamic texture stimuli except for a small uniform spatial region (the scotoma). Following short periods of exposure, observers judged the location of the midpoint between two bars as being biased toward the center of the scotoma and locations near the edge of the scotoma producing the largest shifts in spatial position, consistent with a graded apparent spatial compression. A complication for this approach is that prolonged exposure of artificial scotoma stimuli typically leads to perceptual filling-in, making it difficult to disassociate the effects of adaptation from surface interpolation processes. It is important to note that our use of a narrow annular adapting stimulus precludes this issue—none of the observers in the present study reported perceptual filling in of the annulus in any condition.

The changes in perceived position following exposure to artificial scotomas have been linked to changes in neuronal responses in early visual areas. The response gain and receptive field size of V1 neurons with receptive fields positioned inside the scotoma have been shown to increase (DeAngelis, Anzai, Ohzawa, & Freeman, 1995; Pettet & Gilbert, 1992), potentially reflecting a release from surround suppression (Cavanaugh et al., 2002; Tailby & Metha, 2004). Kapadia et al. (1994) suggest that the increase in receptive field size within the artificial scotoma would have the result that the mean location of active cell receptive fields would be shifted away from its edge. However, this explanation rests on the idea that position is coded by the location of mean activity in a labeled line representation of spatial position, which is undermined by the many examples of motion-induced shifts in apparent location.

The spatial compression effect we report is also similar to that previously described by Hisakata et al. (2016). In their original study, adaptation to an array of dots resulted in two dots presented subsequently in the adapted field to appear closer together. Hisakata and colleagues (2016) attribute their effects to changes in an internal spatial metric, against which judgments of local distance and size are made. However, in their experiments, the test patterns were located in the adapted area, were accompanied by a reduction in perceived texture density, and could occur in the absence of a contextual figural aftereffect. At present, it is difficult to know exactly what process (or combination of processes) underlies the spatial compression observed in the current study. That said, it is clear that this form of spatial distortion is not associated with an increase in crowding.

While all observers in the present study showed a robust spatial compression effect, we found individual differences in the magnitude of the effect and how it varied with stimulus size. For some individuals, the compression approximated a consistent percentage of stimulus size, while others showed a relatively stable absolute reduction in perceived size. In principle, this difference could arise from the adoption of different strategies when judging the size of the stimulus. Hisakata et al. (2016) found that adaptation could introduce an apparent reduction in the size of a Gabor envelope without a concomitant change in the spatial frequency of the carrier. This demonstrates that changes in the scale of global and local aspects of a spatial pattern need not be consistent. If some observers in the present study focused on the local changes in size (i.e., dot separation within each dipole), as opposed to the overall global change in size of the stimulus, this could result in a constant size reduction irrespective of center-flanker distance. Individual differences in overall effect magnitude are comparable to other size related distortions of visual space such as the Ebbinghaus and Ponzo illusions (Grzeczkowski, Clarke, Francis, Mast, & Herzog, 2017; Schwarzkopf & Rees, 2013; Schwarzkopf, Song, & Rees, 2011).

In this paper, we tested to see if orientation crowding is affected by adaptation-induced spatial compression. Our results showed the magnitude of crowding was consistent with the physical but not the perceived location of the flankers. In an additional control experiment, we discounted the possibility that the adapting stimulus produced a uniform scaling of the stimulus, which altered the magnitude of crowding. However, an additional possibility is that the adapting stimulus did not shift the position of all the flankers by a constant magnitude. Crowding is stronger when flankers are aligned horizontally with respect to the target as opposed to vertically (Feng, Jiang, & He, 2007). If the compression of visual space is asymmetric, crowding may remain unchanged even though judgments of overall size are reduced. However, this seems unlikely given observers reported no noticeable change in the shape of the stimulus following adaptation; instead, the adapted stimulus appeared to retain its circular shape, but seemed reduced in size.

Our results contrast with previous studies that have exploited motion-based position shifts to alter apparent stimulus position, where crowding has been shown to vary in accordance with perceived target-flanker separation (Dakin et al., 2011; Maus et al., 2011). This clear difference implies that motion and figural adaptation alter perceived space in fundamentally different ways. One possible reason motion-induced position shifts resulted in altered orientation discrimination thresholds is that they occur at different stages of processing, seemingly with the motion-induced position shift occurring prior to crowding. It is difficult to conclude with any certainty if this is true given the neural locus of crowding itself is a matter of debate. However, it might be argued that judging the orientation of a Gabor patch, a conceivably low-level task, would induce crowding at the early stages of visual processing such as V1 (Millin, Arman, Chung, & Tjan, 2014; Whitney & Levi, 2011), while the motion-induced position shift is associated with higher-level processing in MT (Mather & Pavan, 2009; McGraw, Walsh, & Barrett, 2004). Alternatively, since grouping processes can influence spatial discrimination (Herzog & Manassi, 2015), it is possible that the motion manipulation not only shifts apparent location but also influences grouping. Motion away from the target may degrade the grouping of flankers and target and motion towards the target may increase it. The global spatial compression studied here does not appear to alter the tendency to group targets and distractors.

The more general question of whether the appearance of a stimulus should be associated with visual performance is a longstanding question that dates back to the beginnings of psychophysical research (Fechner, 1860/1966; Ross & Wade, 2010). Initially it may seem intuitive to assume visual performance should follow the perceived properties of the stimulus as though they were physically manipulated, but on closer inspection, this relationship is not trivial. For performance to follow the perceived representation of the stimulus, the ability to perform a task needs to depend on the subjective percept rather than the physical properties of the stimulus. It is clear that performance does not invariably depend upon appearance. Perceptual bias need not affect visual sensitivity, for example geometric illusions, such as the Muller-Lyer and Poggendorff, induce a perceptual bias without a paralleled change in the precision of the representation (Morgan, Hole, & Glennerster, 1990; Tibber, Melmoth, & Morgan, 2008). Solomon and Morgan (2009) make the important point that while concomitant changes in sensitivity can be observed when the perception of a stimulus is distorted, the change in sensitivity may not entirely be due to the distorted perception. The results from the current study further demonstrate that it may not be safe to assume that the perceived properties of a stimulus affect performance in the same way as if they were physically manipulated.

## Figures and Tables

**Appendix A:**
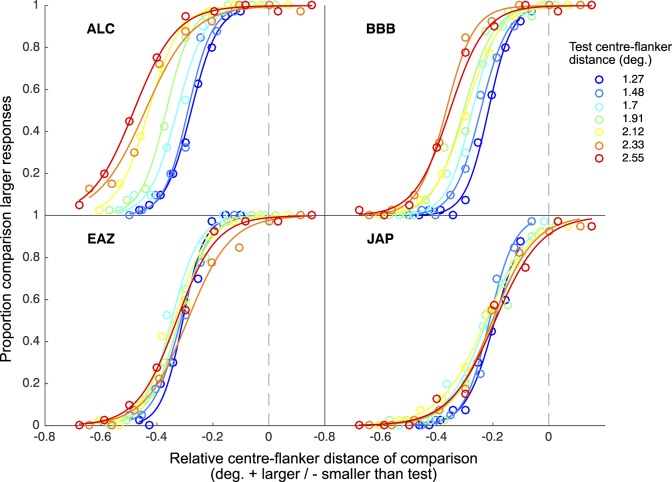
Raw data and psychometric fits for all four observers from the size discrimination experiment. Observers were asked to judge the relative sizes of two stimuli: a “test” stimulus presented at the adapted location and a “comparison” stimulus presented at an unadapted location. The proportion of “comparison larger” responses is plotted as a function of the difference in center-flanker distance between comparison and test stimuli. Separate psychometric functions represent data collected for seven different test center-flanker distances.

**Appendix B:**
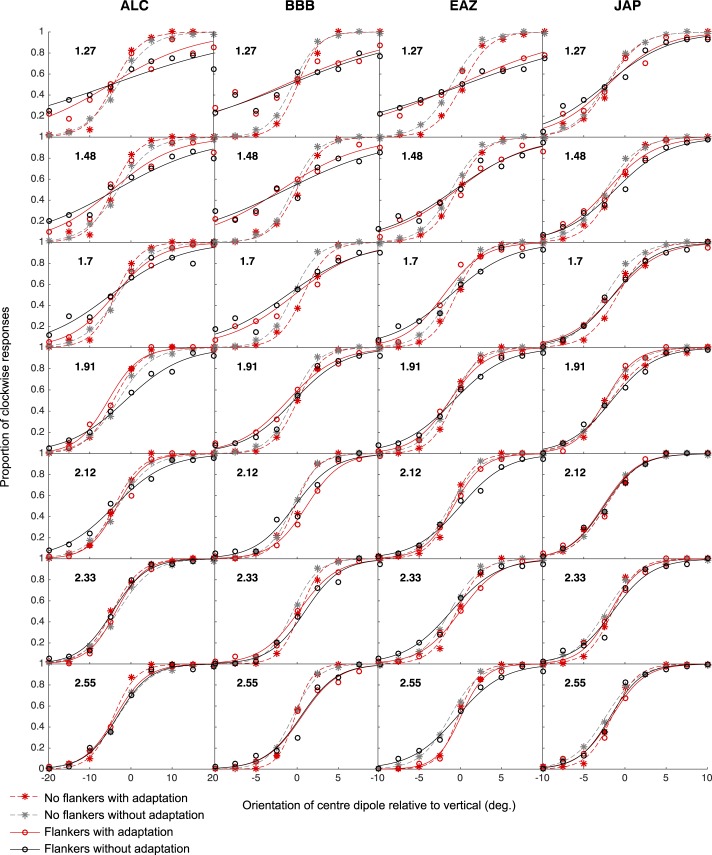
Raw data and psychometric fits for all four observers from the orientation discrimination experiment. In each case, the proportion of clockwise responses is plotted as a function of the orientation of the center dipole relative to vertical. Separate psychometric functions on each plot represent data collected from four different conditions: no flankers/unadapted, flankers/unadapted, no flankers/adapted, and flankers/adapted. Data for separate observers are arranged into columns, whereas data collected at different test center-flanker distances are arranged into rows.
